# Gut publizieren in der Anästhesiologie: Qualitätsstandards und der Umgang mit Predatory Journals

**DOI:** 10.1007/s00101-026-01656-4

**Published:** 2026-02-20

**Authors:** Theresa Tenge, Aileen Hill, David Radke, Götz Schmidt, Justus Student, Laura Borgstedt, Rene M´Pembele, Richard-Felix Kraus, Sebastian Roth, Martin Scharffenberg

**Affiliations:** 1 Klinik für Anästhesiologie, Universitätsklinikum Düsseldorf, Medizinische Fakultät, Heinrich-Heine-Universität, Düsseldorf, Deutschland; 2 Klinik für Anästhesiologie und Klinik für Operative Intensivmedizin und Intermediate Care, Uniklinik RWTH Aachen, Aachen, Deutschland; 3 Klinik für Anästhesiologie und Operative Intensivmedizin, Universitätsklinikum Schleswig-Holstein, Campus Kiel, Kiel, Deutschland; 4 Klinik für Anästhesiologie, operative Intensivmedizin und Schmerztherapie, Justus-Liebig-Universität, Gießen, Deutschland; 5 Klinik für Anästhesiologie und Intensivmedizin, Universitätsklinikum Schleswig-Holstein, Campus Lübeck, Lübeck, Deutschland; 6 TUM School of Medicine and Health, Department Clinical Medicine – Klinik für Anästhesiologie und Intensivmedizin, TUM Klinikum rechts der Isar, Technische Universität München (TUM), München, Deutschland; 7 Klinik für Anästhesiologie, Universitätsklinikum Regensburg, Medizinische Fakultät, Universität Regensburg, Regensburg, Deutschland; 8 Klinik und Poliklinik für Anästhesiologie und Intensivtherapie, Pulmonary Engineering Group, Universitätsklinikum Carl Gustav Carus, Technische Universität Dresden, Fetscherstraße 74, 01307 Dresden, Deutschland

## Hinführung und Relevanz des Themas

Als „traditionelles“ Publikationsmodell werden Veröffentlichungen in Zeitschriften mit Printauflagen bezeichnet, bei denen die Kosten durch die wissenschaftliche Gemeinschaft getragen werden. Das kann durch eine wissenschaftliche Verlagsgesellschaft, Gebühren von Universitäten oder Wissenschaftler*innen oder auch Industriesponsoring erfolgen. Daneben hat sich über die letzten Jahre vermehrt das Open-Access-Modell etabliert, bei dem Autor*innen Article Processing Charges (APC) zahlen und damit Veröffentlichungen für alle frei verfügbar macht [1]. Gleichzeitig gibt es unter den Open-Access-Zeitschriften vermehrt sog. Predatory Journals, die sich nicht an die Prinzipien guter wissenschaftlicher Praxis halten. Predatory Journals sind Zeitschriften und Verlage, die ihre eigenen monetären Interessen auf Kosten der Wissenschaft in den Vordergrund stellen und sich durch falsche oder irreführende Informationen, Abweichungen von bewährten redaktionellen und publizistischen Praktiken, mangelnde Transparenz und/oder aggressive und wahllose Werbepraktiken auszeichnen [2]. Diese Übersichtsarbeit schildert Merkmale dieser Predatory Journals und zeigt praktische Strategien zur Vermeidung auf, um so der Praxis und Wissenschaft in allen Säulen der Anästhesiologie Unterstützung zu bieten.

## Entwicklung von Predatory Journals

Der Begriff Predatory Journals wurde erstmals vom US-amerikanischen Bibliothekar Jeffrey Beall geprägt [3]. Er veröffentlichte ab 2012 auf seinem Blog eine sog. Beall-Liste, in der er Zeitschriften und Verlage auflistete, die durch fragwürdige Praktiken im Bereich des Open Access auffielen. Die Liste wurde von Beall selbst bis 2017 geführt. Kritisiert wurden an der Beall-Liste u. a. die fehlende Aktualität, Unvollständigkeit sowie die mangelnde Transparenz der Kriterien. Darüber hinaus sind nicht alle in der Liste aufgeführten Zeitschriften und Verlage in gängigen wissenschaftlichen Datenbanken auffindbar [1]. Mittlerweile gibt es > 15.000 Predatory Journals, teils wird sogar von mehr als 19.000 berichtet [4]. Auch die Bezeichnung „predatory“ (dt. „räuberisch“) ist nicht unumstritten. Alternativ wurden Begriffe wie „Illegitimate Journals“, „Deceptive Journals“, „Pseudo-Journals“ oder „Dark Journals“ vorgeschlagen. Dennoch hat sich der Begriff Predatory Journals in der wissenschaftlichen Diskussion etabliert und bleibt aufgrund seiner hohen Wiedererkennung im Gebrauch [2].

## Merkmale von Predatory Journals

Insgesamt stehen bei Predatory Journals wirtschaftliche Interessen im Vordergrund [5–7]. Auffällig sind aggressive Werbemaßnahmen, etwa in Form von E‑Mails mit der Aufforderung zur Einreichung von Manuskripten, verbunden mit dem Versprechen einer besonders schnellen Publikation ohne oder mit lediglich kurzen Begutachtungen. Häufig fehlt eine transparente Kostenstruktur, und oft sind die angegebenen wissenschaftlichen Kennzahlen gefälscht oder irreführend. Die Namen dieser Zeitschriften ähneln oft renommierten Journals, was zu Verwechslungen führen kann. Darüber hinaus geben sie häufig fälschlicherweise an, sich an Empfehlungen und Richtlinien von Organisationen wie dem Committee on Publication Ethics (COPE) oder dem International Committee of Medical Journal Editors (ICMJE) zu halten. Auch werden gelegentlich Mitglieder von Editorial Boards genannt, die davon nichts wissen oder nie zugestimmt haben [8]. Diese Praktiken stellen eine ernstzunehmende Bedrohung für die wissenschaftliche Integrität dar. Besonders gefährdet sind junge oder unerfahrene Wissenschaftler*innen, die in der Hoffnung auf schnelle Publikation auf diese Angebote eingehen [9]. Eine Veröffentlichung in einem Predatory Journal kann jedoch die Sichtbarkeit und Rezeption der eigenen Arbeit stark einschränken, die persönliche Reputation schädigen und sich negativ auf Drittmittelanträge oder berufliche Perspektiven auswirken [10].

**Abb. 1:**
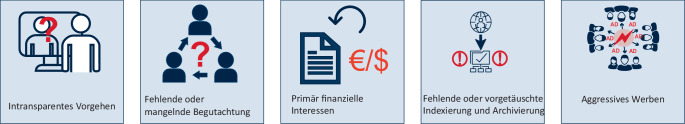
Merkmale von Predatory Journals (© Dr. Laura Borgstedt)

Gängige Merkmale dieser Zeitschriften beinhalten (Abb. 1; [5, 9, 11–14]):

### Intransparentes Vorgehen.

Predatory Journals zeichnen sich durch mangelnde Transparenz in nahezu allen Bereichen aus. Häufig machen sie irreführende Angaben zur Indexierung in renommierten Datenbanken, zur Höhe des Impact Factor, der die durchschnittliche Zitierhäufigkeit von Artikeln einer Zeitschrift innerhalb eines bestimmten Zeitraums angibt, oder zur Zusammensetzung des Editorial Board. Die Zeitschriftentitel klingen oft bedeutungsvoll („World Journal of …“) oder imitieren etablierte Zeitschriften und Verlage, um Seriosität vorzutäuschen. Angaben zu APC sind oft unvollständig, schwer auffindbar oder werden erst nach Annahme des Manuskripts genannt. In einigen Fällen wird die Zahlung der APC sogar bereits vor einer Begutachtung gefordert. Zudem fehlt es oft an klaren Informationen zu Fristen, Kostenstrukturen oder Bewertungsmaßstäben.

### Fehlende oder mangelnde Begutachtung.

Ein zentrales Merkmal ist das Fehlen eines echten Peer-Review-Verfahrens. Stattdessen erfolgt die Annahme oft automatisiert, ohne inhaltliche Prüfung oder nach extrem kurzer Bearbeitungszeit. Manuskripte werden teilweise innerhalb weniger Tage akzeptiert, ohne dass substanzielle Änderungen gefordert werden. Die Nutzung von künstlicher Intelligenz (KI) wie ChatGPT oder anderen KI-Alternativen ist vermutlich ebenfalls präsent.

### Website und Veröffentlichungsstandards.

Die Webseiten von Predatory Journals wirken durch veraltete Gestaltung, fehlerhafte Layouts oder sprachliche Mängel häufig unprofessionell. Auch die publizierten Artikel zeigen häufig Anzeichen mangelnder redaktioneller Bearbeitung. Zentrale Informationen zu redaktionellen Richtlinien, zum Vorgehen der guten wissenschaftlichen Praxis (retraction, corrigendum/erratum) oder klare Kontaktinformationen fehlen oder sind nicht überprüfbar. In manchen Fällen werden Artikel sogar kommentarlos entfernt.

### Finanzielle Interessen.

Das primäre Ziel dieser Zeitschriften ist der finanzielle Gewinn, nicht die Verbreitung wissenschaftlicher Erkenntnisse. Auch wissenschaftlich irrelevante oder qualitativ mangelhafte Arbeiten werden veröffentlicht, sofern die APC bezahlt werden. Das Open-Access-Modell wird dabei missbraucht: Zwar wird eine Veröffentlichung gegen Gebühr versprochen, jedoch ohne die zugehörigen publizistischen Leistungen wie Qualitätssicherung oder dauerhafte Archivierung zu gewährleisten.

### Indexierung und Archivierung.

Viele Predatory Journals behaupten fälschlich, in renommierten Datenbanken wie PubMed oder Scopus indexiert zu sein. Tatsächlich lassen sich ihre Publikationen dort teils nicht finden. Auch fehlen oftmals standardisierte Archivierungsverfahren, eindeutige und dauerhafte Identifikatoren (Digital Object Identifier, DOI) oder eine langfristige Zitierbarkeit. Angaben zu journal metrics oder impact factors sind teils gefälscht oder frei erfunden.

### Aggressives Werben.

Ein weiteres typisches Merkmal ist das wiederholte und aufdringliche Werben per E‑Mail. Wissenschaftler*innen werden mit schmeichelnden Nachrichten zur Einreichung von Manuskripten oder zur Mitarbeit im editorial board eingeladen. Diese E‑Mails enthalten oft sprachliche Fehler sowie unrealistische Versprechungen zu Sichtbarkeit, Impact Factor oder Indexierung.

## Predatory Journals im AINSP-Bereich

Auch der Bereich der Anästhesiologie, Intensiv‑, Notfall‑, Schmerz- und Palliativmedizin (AINSP) bleibt von Predatory Publishing nicht verschont. Eine gezielte Suche nach verschiedenen AINSP-bezogenen Stichworten in einer öffentlich zugänglichen Kopie der Beall-Liste (Stand: Dezember 2024), die inzwischen von einem anonymen Hoster gepflegt und fortlaufend ergänzt wird, ergab jedoch nur begrenzte Treffer mit vier Zeitschriften. Unter dem Suchterminus „Surg“ finden sich neun Zeitschriften, unter „medic“ 15 Verlage und 144 Zeitschriften. Die begrenzte Trefferzahl für AINSP könnte auf zwei Aspekte hinweisen: Einerseits ist die Beall-Liste in ihrer aktuellen Form nur eingeschränkt aktuell und vollständig, da neue Predatory Journals kontinuierlich hinzukommen. Andererseits stellt sich die Frage, ob Fachzeitschriften aus dem AINSP-Bereich für die eher allgemein gehaltene Beall-Liste zu spezifisch und damit unterrepräsentiert sind. Zwei weiterführende Analysen von Cortegiani et al. aus dem Jahr 2019 zeigen allerdings, dass in der Anästhesiologie 212 potenzielle Predatory Journals von 83 Verlagen (davon sechs in PubMed gelistet) und in der Intensivmedizin 86 Zeitschriften von 48 Verlagen (davon drei mit PubMed-Indexierung) identifiziert werden können [10, 15]. Auffällig war der Mangel an grundlegenden Qualitätsmerkmalen: 42 %/43 % (Anästhesiologie/Intensivmedizin) der Zeitschriften verfügten über keine überprüfbare Adresse, nur 32 %/29 % benannten eine(n) Editor-in-Chief, und in 79 %/81 % war das Editorial Board zwar aufgeführt, aber oft nicht verifizierbar. Auch die finanziellen Strukturen waren problematisch: Die medianen APC lagen bei 635 bzw. 910 US-Dollar in der Anästhesiologie und Intensivmedizin. Zudem wurden häufig fragwürdige oder gefälschte Journal Metrics genutzt, um den Eindruck wissenschaftlicher Seriosität zu erwecken.

## Maßnahmen zur Bekämpfung von Predatory Journals

Besonders gefährdet, auf Predatory Journals hereinzufallen, sind junge Wissenschaftler*innen am Beginn ihrer Karriere, insbesondere wenn sie nur unzureichend betreut oder beraten werden. Der zunehmende Publikationsdruck, gepaart mit fehlender Erfahrung im wissenschaftlichen Publikationsprozess, erhöht das Risiko zusätzlich [8]. Neben der Beall-Liste mit ihren genannten Limitationen gibt es keine zentrale, zuverlässige Blacklist für unseriöse Zeitschriften und Verlage. Umfassende oder wirksame Kampagnen gegen die Betreiber von Predatory Journals existieren derzeit ebenfalls nicht, und juristische Maßnahmen verlaufen oft im Sande, da die hinter den Zeitschriften stehenden Organisationen häufig schwer greifbar sind, anonym agieren und nicht auf Anfragen oder Beschwerden reagieren [8]. Der Fokus liegt daher verstärkt auf Präventionsmaßnahmen, um das Einreichen bei Predatory Journals von vornherein zu vermeiden. Dazu zählen sog. Whitelists, etwa das Directory of Open Access Journals (DOAJ; https://doaj.org/) oder interne Empfehlungen von Universitäten, auf die sich Forschende bei der Auswahl geeigneter Zeitschriften und Verlage stützen können [3, 16, 17]. Auch die Deutsche Forschungsgemeinschaft und andere Leitfäden geben Wissenschaftler*innen Hilfestellungen [18, 19]. Eine wichtige Initiative ist zudem die Plattform „Think. Check. Submit.“ (https://thinkchecksubmit.org/), die durch eine interdisziplinäre Zusammenarbeit verschiedener wissenschaftlicher Verbände und Organisationen ins Leben gerufen wurde [20]. Das Ziel ist es, bei der Auswahl seriöser Zeitschriften zu unterstützen [21]. Weitere Ressourcen sind die ICMJE-Empfehlungen sowie das gemeinsame Statement „Principles of Transparency and Best Practice in Scholarly Publishing“ verschiedener internationaler Organisationen [22, 23]. Weitere Hilfsmittel sind das kostenpflichtige Verzeichnis Cabell’s Predatory Reports (cabells.com) sowie Angebote einzelner Universitäten, etwa der Universitätsbibliothek Utrecht, die umfangreiche Beratung und Tools zum Erkennen von Predatory Journals bereitstellt [24]. Ergänzend gibt es eine neuere, open-source-basierte Plattform (predatoryjournals.org), die von anonymen Wissenschaftler*innen gepflegt wird, die nach eigenen Angaben selbst negative Erfahrungen mit Predatory Journals gemacht haben. Dessen Qualität und Neutralität werden jedoch derzeit noch kritisch diskutiert.

## Gute Publikationspraxis

### Auffinden von guten Zeitschriften und Verlagen

Die Wahl einer geeigneten Zeitschrift ist ein wichtiger Schritt im Publikationsprozess. Allerdings erschwert die nahezu unüberschaubare Vielzahl der Fachzeitschriften, die sich in ihren Schwerpunkten, Qualitätsstandards und Reichweiten mitunter maßgeblich unterscheiden, die Auswahl. Bei der Suche nach dem Ziel-Journal können verschiedene Herangehensweisen hilfreich sein, wie beispielsweise der Blick in die verwendeten Quellen: Thematisch verwandte Arbeiten erscheinen oft in denselben Zeitschriften, die damit naheliegende Kandidaten darstellen. Ebenso können Fachzeitschriften, in denen führende Autor*innen regelmäßig publizieren, eine wertvolle Orientierung bieten. Das sorgfältige Prüfen der Angaben der Zeitschriften und Verlage zu „Aims and Scope“, ihre Indexierung und ihr Impact Factor können zusätzlich hilfreich sein. Schließlich kann die Diskussion mit erfahrenen Kolleg*innen die Entscheidung absichern und helfen, die Erfolgschancen abzuschätzen. Um Forschende bei der Orientierung zu unterstützen, wurden sog. Journal Finder entwickelt. Diese Online-Tools schlagen, basierend auf Angaben wie Titel, Abstract oder Keywords, passende Zeitschriften vor und bieten häufig Zusatzinformationen zu Impact Factor, Indexierung oder Open-Access-Optionen. Mit wenigen Eingaben, die von Algorithmen mit den Profilen der Zeitschriften und Verlage abgeglichen werden, lassen sich so thematisch passende Journals identifizieren, die anschließend hinsichtlich Sichtbarkeit, durchschnittlicher Zeit von Einreichung bis zur Veröffentlichung oder Leserschaft verglichen werden können. Einige Tools stehen kostenlos zur Verfügung, andere sind kostenpflichtig. Allerdings sollten die Vorschläge kritisch bewertet werden. Viele solcher Angebote sind verlagsspezifisch und zeigen entsprechend Zeitschriften des jeweiligen Anbieters, wodurch andere Optionen übersehen werden können. Auch basiert die Auswahl meist auf Textähnlichkeiten, ohne die inhaltliche Tiefe oder wissenschaftliche Relevanz vollständig abzubilden. Aspekte wie Reputation, Leserschaft oder redaktionelle Ausrichtung lassen sich nicht automatisiert erfassen. Häufig werden keine passenden Journals vorgeschlagen, wenn die Keywords zu spezifisch sind. Journal Finder sind daher als Orientierungshilfe, nicht aber als alleinige Entscheidungsgrundlage zu verstehen. Sie bieten eine schnelle und strukturierte Übersicht, ersetzen jedoch nicht die kritische Auseinandersetzung mit dem Publikationsumfeld. Im Idealfall werden die Vorschläge mit zusätzlicher Literaturrecherche und fachlichem Rat kombiniert, um die Chancen auf eine erfolgreiche und sichtbare Veröffentlichung zu maximieren. Tab. 1 gibt einen Überblick über verschiedene Journal-Finder und Tools.

**Tab. 1:** Auswahl verschiedener Journal Finder

Anbieter	Tool	Link	Kosten-modell
*Elsevier*	Journal Finder	https://journalfinder.elsevier.com/	Kostenlos
*Wiley*	Journal Finder	https://journalfinder.wiley.com/	Kostenlos
*Springer Nature*	Journal Suggester	https://journalsuggester.springer.com/	Kostenlos
*SAGE*	Journal Recommender	https://journal-recommender.sagepub.com/	Kostenlos
*Taylor & Francis*	Journal Suggester	https://authorservices.taylorandfrancis.com/publishing-your-research/choosing-a-journal/journal-suggester/	Kostenlos
*LWW (Lippincott Williams & Wilkins)*	Journal Finder	https://editingservices.lww.com/journal-finder	Kostenlos
*JANE*	Journal/Author Name Estimator	https://jane.biosemantics.org/	Kostenlos
*JournalGuide*	Journal Matching	https://www.journalguide.com/	Kostenlos
*DOAJ*	Directory of Open Access Journals	https://doaj.org/	Kostenlos
*Clarivate (Web of Science)*	Manuscript Matcher	https://mjl.clarivate.com/manuscript-matcher	Kostenpflichtig (Lizenz nötig)
*EndNote (Clarivate)*	Manuscript Matcher in EndNote	https://endnote.com/product-details/manuscript-matcher/	Kostenpflichtig (EndNote Lizenz)
*Cabells*	Journalytics & Predatory Reports	https://www.cabells.com/	Kostenpflichtig (Abo)
*Edanz*	Journal Selector	https://www.edanz.com/journal-selector	Basis kostenlos, Pro kostenpflichtig

Eine mögliche Vorgehensweise bei der Auswahl des Journals könnte sein:

1. Identifikation möglicher Ziel-Journals mittels kostenlosem Journal-Finder.
2. Prüfung der Qualität der Fachzeitschriften und Verlage über z. B. DOAJ, PubMed oder Web of Science und Whitelists, um offensichtliche Predatory Journals zu vermeiden.
3. Evaluation des Ziel-Journals anhand von:
  Homepage vorhanden?
  Impressum vorhanden?
  Adresse existierend?
  Namentlich genannter Editor-in-Chief?
  Namentlich genanntes Editorial Board?
  Angaben zur durchschnittlichen Dauer von Einreichung bis zur Veröffentlichung?
  APC, inklusive Summe, genannt?
  Angaben und Prüfung von Indexierung (PubMed, Scopus etc.)?
  Angaben zum Impact Factor (oder anderen Metriken)?
  Publikationsaktivitäten?
4. Vergleiche zu Referenzen in den jeweiligen Forschungsfeldern: Was wird in den bedeutsamen Publikationen zitiert?

### Journal Citation Reports

Eine weitere Möglichkeit zur Auswahl eines geeigneten Journals stellen die Journal Citation Reports (JCR) des Unternehmens Clarivate dar [25]. Es handelt sich dabei um eine umfassende Datenbank zur Bewertung und zum Vergleich wissenschaftlicher Fachzeitschriften. Sie basieren auf den im Web of Science indexierten Journals und bieten somit eine qualitätsgesicherte Auswahl seriöser wissenschaftlicher Publikationsorgane. Die Aufnahme einer Zeitschrift in den JCR gilt als Indikator für wissenschaftliche Integrität und Relevanz, da sog. Predatory Journals ausgeschlossen werden.

Im Rahmen der JCR werden verschiedene bibliometrische Kennzahlen bereitgestellt, die der Beurteilung der wissenschaftlichen Wirkung einer Zeitschrift dienen. Neben dem Impact Factor werden weitere Indikatoren, wie etwa der Immediacy Index oder die Cited Half-life, berücksichtigt. Auf Grundlage dieser Kennzahlen erstellt Clarivate Rankings innerhalb einzelner Fachgebiete, die den Vergleich und die Einordnung der wissenschaftlichen Relevanz verschiedener Zeitschriften ermöglichen. Für die Anästhesiologie gibt es im JCR eine eigene Kategorie, die hilfreich sein kann, um ein geeignetes Journal für einen spezifischen Artikel zu finden.

## Transparenter Umgang mit künstlicher Intelligenz im Peer-Review-Prozess

Künstliche Intelligenz dringt mittlerweile in viele Bereiche der Wissenschaft vor; nicht nur das Erstellen von wissenschaftlichen Artikeln, auch der Peer-Review-Prozess wird durch KI beeinflusst.

Angesichts der zunehmenden Verbreitung generativer KI-Tools in Peer-Review-Prozesse haben wissenschaftliche Verlage und relevante Institutionen begonnen, Vorschriften für die Verwendung solcher Tools für den Peer-Review-Prozess zu erlassen. Am 23. Juni 2023 haben die National Institutes of Health (NIH) ein Verbot für die Verwendung von generativen KI-Tools wie ChatGPT für die Analyse und Erstellung von Peer-Review-Kommentaren eingeführt. Auch der Australian Research Council (ARC) verbot die Verwendung generativer KI bei Peer Reviews. Was Zeitschriften betrifft, so empfehlen die neuesten Empfehlungen des ICMJE, dass Gutachter keine Manuskripte hochladen sollten in KI-Software, die eine Vertraulichkeit nicht gewährleisten können. Namhafte medizinische Journals (wie etwa *The Lancet, Science oder JAMA*) verbieten die Verwendung großer Sprachmodelle während des Peer-Review-Prozesses ganz und untersagen es Gutachter*innen, Manuskripte in generative KI-Tools hochzuladen, weil es gegen die Vertraulichkeitsrichtlinien verstoße [26].

Dieser komplette Bann der KI vom Peer-Review-Prozess ist jedoch umstritten und eine differenzierte Vorgehensweise wird gefordert [27]. So gibt es Stimmen, dass Gutachter*innen die Möglichkeit gegeben werden sollte, ein KI-Tool in einer Weise zu verwenden, die nicht gegen die Vertraulichkeitsrichtlinien der Zeitschrift verstößt. Dann müssen jedoch Name und Verwendungsweise des Tools angegeben werden [26, 28, 29]. In jedem Fall bringt der Einsatz generativer KI eine Vielzahl ethischer und rechtlicher Probleme mit sich, darunter Fragen des Datenschutzes und des Urheberrechtsschutzes. Diese Themen erfordern eine umfassende Prüfung und Diskussion im Rahmen des Peer-Review-Prozesses, nicht zuletzt um die Qualität des Peer-Review-Prozesses aufrechtzuerhalten [27]. Zudem ist eine uneingeschränkte Transparenz nötig, um eine eindeutige Abgrenzung von Predatory Journals auch in diesem Punkt erzielen zu können.

Im AINSP-Bereich gibt es jedoch leider eine große Lücke für Vorgaben zur Verwendung von KI-Tools bei der Erstellung von Peer-Review-Gutachten. Cheng et al. berichteten 2024 in *Critical Care*, dass die zehn renommiertesten Fachzeitschriften mit dem höchsten Impact Factor in diesem Bereich (mit Ausnahme von *Lancet Respiratory Medicine*) keine Angaben zu KI und KI-gestützten Technologien im Peer-Review-Prozess machten. Es erscheint daher dringend notwendig, dass die Fachzeitschriften hier nachbessern und ihre Richtlinien zur Verwendung von KI-Tools im Peer-Review-Prozess umgehend aktualisieren [26].

Ähnlich wie Verlage KI als selbstständige Autoren ausschließen, sollten sie auch vollständig autonome Peer Reviews verbieten, da KI nicht für ihre Ergebnisse haftbar gemacht werden kann. Eine überwachte Nutzung ist aus Sicht der Autor*innen jedoch akzeptabel, wenn sie deklariert wird. Diese Richtlinien können auch die Transparenz zwischen Gutachter*innen, Autor*innen, Herausgeber*innen, Leser*innen und der Gesellschaft fördern, einen verlässlichen Peer-Review-Prozess begünstigen und die Abgrenzung zu Predatory Journals erleichtern [28].

### Infobox WAKWiN

Das Ziel des Wissenschaftlichen Arbeitskreises Wissenschaftlicher Nachwuchs (WAKWiN) der Deutschen Gesellschaft für Anästhesiologie und Intensivmedizin (DGAI) ist die Förderung und wissenschaftliche Ausbildung junger Anästhesiolog*innen. Durch vielfältige regelmäßige Veranstaltungen und das Mentoring-Programm fördert der WAKWiN überregionale Vernetzung sowie die professionelle und persönliche Entwicklung akademisch Interessierter verschiedener Karrierestufen in allen Säulen der Anästhesiologie. Nähere Informationen s. www.wakwin.dgai.de.

## Fazit für die Praxis

- Predatory Journals stellen eine ernstzunehmende Herausforderung für die wissenschaftliche Integrität dar. Sie lassen etablierte Publikationsstandards außer Acht, verfolgen primär finanzielle Interessen und können besonders Nachwuchswissenschaftler*innen, die unter hohem Publikationsdruck stehen, schaden.
- Obwohl es Warnlisten wie die Beall-Liste gibt, fehlt es bislang an einer verlässlichen, umfassenden Blacklist. Der Fokus liegt daher auf Prävention mittels institutioneller Leitlinien, Whitelists sowie internationaler Initiativen.
- Eine bewusste und verantwortungsvolle Auswahl des Journals sowie gezielte Aufklärung über Predatory Journals sind zentrale Maßnahmen, um die Verbreitung pseudowissenschaftlicher Publikationen einzudämmen und wissenschaftliche Qualität langfristig zu sichern.
